# Digital and Analog Resistive Switching Behavior in Si-NCs Embedded in a Si/SiO_2_ Multilayer Structure for Neuromorphic Systems

**DOI:** 10.3390/nano13060986

**Published:** 2023-03-09

**Authors:** Alfredo Morales-Sánchez, Karla Esther González-Flores, Sergio Alfonso Pérez-García, Sergio González-Torres, Blas Garrido-Fernández, Luis Hernández-Martínez, Mario Moreno-Moreno

**Affiliations:** 1 Electronics Department, Instituto Nacional de Astrofísica, Óptica y Electrónica, Puebla 72840, Mexico; luish@inaoep.mx (L.H.-M.); mmoreno@inaoep.mx (M.M.-M.); 2 Centro de Investigación en Materiales Avanzados S.C., Unidad Monterrey, Parque de Investigación e Innovación Tecnológica (PIIT), Apodaca 66628, Nuevo León, Mexico; alfonso.perez@cimav.edu.mx; 3 MIND, Department of Electronics and Biomedical Engineering, Universitat de Barcelona, Martí i Franquès 1, E-08028 Barcelona, Spain; s.gonzalez.torres@ub.edu (S.G.-T.); blas.garrido@ub.edu (B.G.-F.)

**Keywords:** silicon nanocrystals, multilayers, resistive switching, memristors, neuromorphic computing

## Abstract

In this work, we report the digital and analog resistive-switching (RS) characteristics in a memristor based on silicon nanocrystals (Si-NCs) integrated into a complementary metal-oxide-semiconductor (MOS) structure. Si-NCs with a diameter of 5.48 ± 1.24 nm embedded in a SiO_2_/Si-NCs/SiO_2_ multilayer structure acts as an RS layer. These devices exhibit bipolar RS with an intermediate resistance step during SET and RESET processes, which is believed to lie in the Si-NCs layer acting as charge-trapping nodes. The endurance studies of about 70 DC cycles indicate an ON/OFF ratio of ~10^6^ and a retention time larger than 10^4^ s. Long-term potentiation (LTP, −2 V) and long-term depression (LTD, +4 V) are obtained by applying consecutive identical pulse voltages of 150 ms duration. The current value gradually increases/decreases (LTP/LTD) as the pulse number increases. Three consecutive identical pulses of −2 V/150 ms (LTP) separated by 5 and 15 min show that the last current value obtained at the end of each pulse train is kept, confirming an analog RS behavior. These characteristics provide a possible way to mimic biological synapse functions for applications in neuromorphic computing in Si-NCs-based CMOS structures.

## 1. Introduction

The conventional digital architecture based on the Von-Neumann architecture faces various challenges as computational complexity increases, due to the inefficient transfer of data between separated processing units and memories [1,2]. As a possible solution, neuromorphic computing, inspired by the characteristics of biological brains, has attracted considerable attention [1,3,4]. Memristors, which are non-volatile memory elements based on the resistive-switching (RS) phenomenon, exhibit promising characteristics in developing artificial synapses for implementing brain-inspired neuromorphic computing, due to their low energy consumption and high efficiency [5,6,7,8,9,10]. A memristor can store information in the form of resistance, typically with an abrupt switching between a high resistance state (HRS) and a low resistance state (LRS), i.e., digital switching [2,10]. Memristors with analog states, however, are particularly interesting due to their ability to express gradual changes in the resistance state [11,12]. In an analog RS device, the resistance can be gradually changed depending on the number of voltage pulses applied, which is important to mimic the biological synapse. The development of digital or analog memristive devices is of great interest. Nevertheless, memristor devices showing both analog and digital RS behaviors would allow one to reduce the complexity of the circuitry. Materials exhibiting both RSs have been observed to depend on the bias voltage, thickness or a change in the electrode [13,14,15,16]. Transition metal oxides such as WO_3_, Nb_2_O_5_, TaO_x_ and HfO_x_ are the most common type of active materials in analog RS memristors [8,17,18,19,20]. Additionally, the combination of the advantages of resistive random-access memory and a ferroelectric field-effect transistor has been reported in Hf_0.5_Zr_0.5_O_2_ films for its application to high-accuracy on-chip deep neural networks [21]. Nevertheless, to make RS devices more attractive for technology transfer and integration, the active materials should be compatible with the complementary-metal-oxide-semiconductor (CMOS) technology due to its low production cost. A material that offers such a compatibility is silicon rich oxide (SRO or SiO_x_, x < 2). Recently, SRO films containing Si-nanocrystals (Si-NCs) have been widely studied for application in RS devices [22,23,24,25]. A demonstration of the experimental implementation of synaptic functions in nanoscale silicon-based memristors was proposed by [26]. In these devices, the filament was related to the presence of Ag, since they used the combination of layers with Ag-rich (high conductivity) and Ag-poor (low conductivity) regions. Some other studies reporting digital and analog RS behavior are based on the use of Pt dispersed silicon oxide-based memristors and by combining a buffer layer of TiO_x_ with a SiO_x_:Ag layer, respectively [27,28]. Again, in these devices the RS is related to the formation and annihilation of a metallic-based conductive filament.

Pure SiO_x_-based nano-memristors, produced by a novel nanoembedding-and-oxidation-of-nanoparticles procedure, have shown a regeneration of RS properties after 1000 cycles by the application of a positive voltage higher than +15 V for 500 ms, which establishes again the condition for Si oxidation [29,30]. At the same time, the electrical characterization of SRO films clearly shows not only an intrinsic RS behavior but also the presence of multiple conductance states [31,32]. In our previous works, we demonstrated the control of unipolar and bipolar abrupt (i.e., digital) RS for Si-NCs embedded in Si/SiO_2_ multilayer (ML) structures [32,33]. These devices exhibit multilevel RS behavior, that is, different resistance states during the partial formation and annihilation of the conductive filaments. Furthermore, the number of these intermediate states can be tuned by changing the number of Si-NCs layers in the ML, which also allows for the fine control of other important device parameters, including SET and RESET voltages.

In this work, we introduce a Si-NCs-based memristor that exhibits both digital and analog switching, a property that has been seldom reported for this material. Si-NCs with a diameter of 5.48 ± 1.24 nm were formed in the SiO_2_/Si-NCs/SiO_2_ multilayer structure after thermal annealing. The digital RS indicates that these devices exhibit an ON/OFF ratio of ~10^6^ and a retention time larger than 10^4^ s. Meanwhile, the analog response of the memristor devices based on Si-NCs was analyzed by using identical voltage pulses. Compared to complex programming schemes, such as the variation of the voltage amplitude, pulse length, or current compliance, the use of identical pulses greatly reduces the complexity of the pulse-driving circuit, a critical requirement for their future application. Long-term potentiation (LTP, −2 V) and long-term depression (LTD, +4 V) are obtained by applying consecutive identical pulse voltages of 150 ms duration. Our results reveal a gradual change in electrical resistance for both the SET and RESET processes that can be controlled by the number of applied pulses.

## 2. Materials and Methods

**Figure 1:**
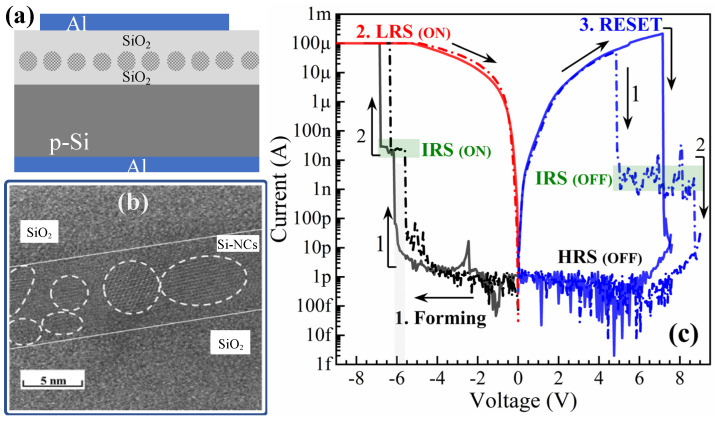
(**a**) Schematic of the MOS-like structure (not at scale); (**b**) cross-sectional TEM image of the SiO_2_/Si-NCs/SiO_2_ device; and (**c**) typical I-V curves of bipolar resistive switching. During forming and RESET process, an intermediate resistive state (IRS) is observed.

MOS-like structures were fabricated using a SiO_2_/Si-NCs/SiO_2_ ML as RS material and deposited on a p-type (100) silicon substrate with a 1–5 Ωcm resistivity, with an Al electrode as the top contact, as schematized in Figure 1a. First, the SiO_2_/Si/SiO_2_ ML was deposited with an RF magnetron sputtering system by Torr International^TM^. The ML process alternates the deposition of SiO_2_ and Si layers by the sputtering of pure SiO_2_ and Si targets in an argon plasma at 500 °C. The thickness of SiO_2_/Si/SiO_2_ layers was ~15 nm, ~6 nm and ~7 nm, respectively. The Si substrate was cleaned before deposition using an ultrasonic bath with acetone, ethanol, and deionized water, followed by submersion in an aqueous solution of hydrofluoric acid (HF). Then, after deposition, the MLs were thermally annealed at 1100 °C for 1 h under N_2_ atmosphere in a quartz tube furnace to promote the Si-NCs’ formation. The internal structure of the SiO_2_/Si-NCs/SiO_2_ ML system and successful formation of Si-NCs was confirmed by a high-resolution transmission electron microscopy (HRTEM) analysis using a microscope JEOLTM JEM 2200 (Jeol Ltd., Tokyo, Japan). Aiming to analyze the electric behavior of the devices, an Al electrode was deposited over the SiO_2_ barrier layer using an electron beam evaporation (e-beam) system. The gate was defined by standard photolithography and lift-off processes with square patterns, with an area of 250 µm × 250 µm. Al Ohmic contacts were deposited on the back side of the substrates by electron beam evaporation at room temperature with a thickness of ~300 nm. Finally, the devices were thermally annealed at 460 °C for 20 min to improve the ohmic contact. The current-voltage (I-V) characteristics were measured using an Agilent Technologies B1500A semiconductor device parameter analyzer. Finally, for analog RS measurements, a B1525A high-voltage semiconductor pulse generator unit (HV-SPGU) installed on the B1500 was utilized. All experiments were performed in ambient conditions.

## 3. Results

Figure 1b shows a cross-sectional view of the TEM micrograph of the SiO_2_/Si-NCs/SiO_2_ ML using a bright field detector (BF). As we can see, the ML structure allows for the formation of Si-NCs confined in the Si layer between the SiO_2_ layers. Moreover, the micrograph recorded with the BF detector reveals the successful formation of Si-NCs, which are evidenced by the marked different orientations of their atomic planes. The diameter of Si-NCs obtained in the ML structure is about 5.48 ± 1.24 nm.

Figure 1c shows the typical I-V curves of two different devices with the SiO_2_/Si-NCs/SiO_2_ ML structure. Both devices show the same two-step forming process at forward bias (FB, black lines), in which a first abrupt current step occurs at −6.15 V (continuous line), acting as an intermediate resistive switching (IRS) state, followed by a second step at −6.85 V. The first step (IRS) shows self-compliance, consistently stopping at around 20 nA across devices. For the second step, however, the current compliance (CC) is typically set at 100 μA to limit its maximum current. After the electroforming process, the devices show a bipolar resistive switching behavior, with a negative bias inducing the SET process (HRS to LRS) and a positive bias triggering the RESET process, returning to the HRS. Two kinds of RESET processes can occur: an abrupt RESET (continuous line) from LRS to HRS, and a step-wise RESET (dash-dot line), akin to the two-step SET discussed above. Multiple steps observed in the I-V curve represent different SETs before obtaining the highest current value reached in the devices, which is the partial formation of the conductive filament in the SiO_2_/Si-NCs/SiO_2_ ML structure. For the RESET process, one observes a voltage range (step-wise, 4.9 V–8.7 V) where the current increases (jumps) and decreases (drops), which is IRS (OFF). These effects can be related to a possible competition of the re-formation (jumps) and partial rupture (drops) until the complete annihilation of the conductive filament. Based on the conductive filament’s nature, if this is strong enough, it will require a higher energy to be destroyed, which is reached with a higher voltage and, in some cases, without the observation of the IRS (abrupt, at 7.2 V). In fact, in a previous work, we reported a multilevel bipolar RS behavior in structures with eight Si-NCs/SiO_2_ bilayers [32]. In these structures, the resistance level of the device is gradually switched through several IRSs, controlled by the stop voltage during the continuous DC sweep in the electroforming, SET and RESET processes. The origin of these IRSs (stair-like current) has been attributed to Coulomb blockade effects in Si-NCs [34,35,36], which act (in our devices) as charge trapping nodes. In contrast to these studies, in this work we analyze a structure of one Si-NCs layer confined between two SiO_2_ layers, which shows one intermediate state. This single intermediate level first corroborates the relation between the number of current steps and the Si-NCs layers, and second, it represents the partial formation/rupture of the conductive filament, connecting/disconnecting the Si-NCs layer with the neighboring SiO_2_ layers.

**Figure 2:**
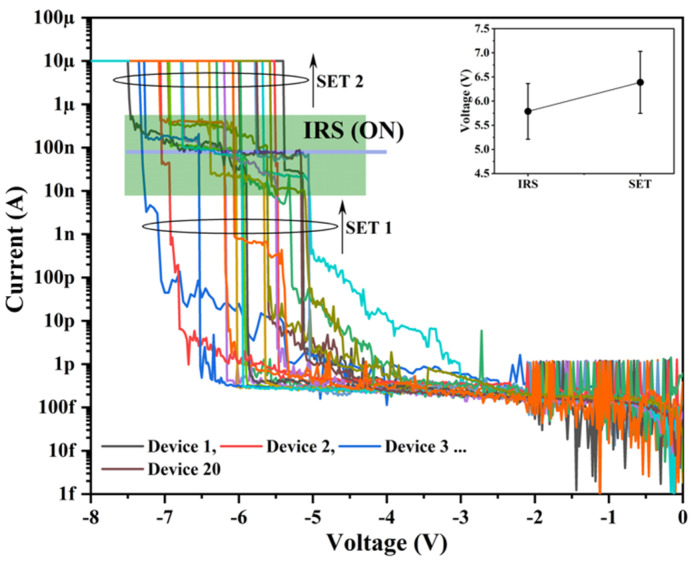
Device-to-device variation of I-V curves in the electroforming process. Inset shows the voltage at which the two-step forming process occurs, IRS (SET 1) and SET 2. Different color lines indicate the I-V curves (electroforming) of 20 devices.

Figure 2 shows the I-V curves of 20 different devices to observe the device-to-device variation in the electroforming process. As we can observe, the voltage at which the IRS (SET 1) and SET 2 occur is about 5.78 ± 0.57 V and 6.38 ± 0.64 V (see inset), respectively. The variation is about 10% for both SET 1 and SET 2. Additionally, the current value of the IRS ranges from 10 nA to 412 nA (green zone). Nevertheless, the same two-step forming process is observed for all of the tested devices (20 devices). The variation of these voltages from device to device can be attributed to the stochastic nature of the formation of the conductive filament. Moreover, the sputtering deposition process typically forms columnar or vertical grains that act as potential sites for multiple conductive filament formation, allowing for the variation of the voltages of IRS and SET 2.

**Figure 3:**
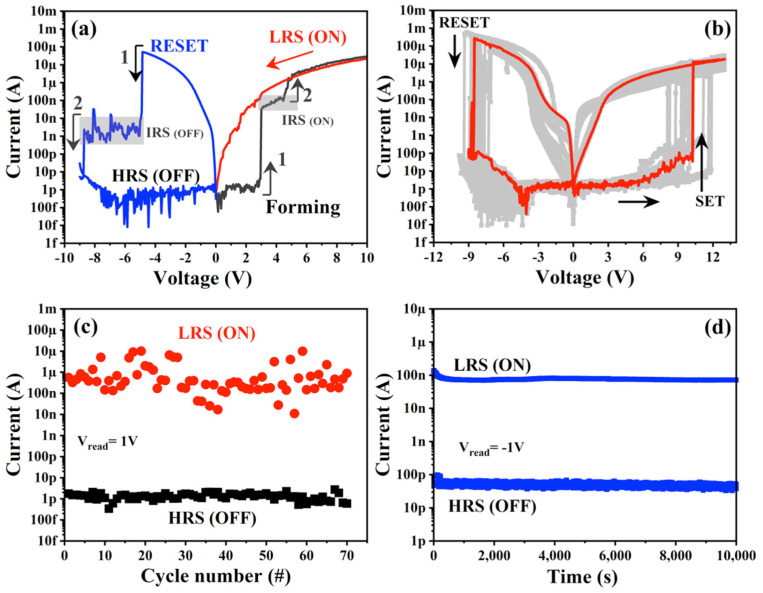
(**a**) Resistive switching: typical I-V curves at RB, (**b**) DC I-V cycles, (**c**) endurance (V_read_ = 1 V) and (**d**) retention time (V_read_ = −1 V).

The electroforming process was also analyzed by biasing the devices in reverse bias (RB), as shown in Figure 3a. As we can observe, the same two-step forming process is obtained, in which a first abrupt step occurs at 3 V, followed by a second step at 4.5 V. Under RB, the CC was not required because of devices’ self-compliance. These devices, electroformed in RB, also exhibit a step-wise RESET, akin to the two-step SET. Different I-V cycles were measured, after electroforming in RB, to obtain the endurance (without CC) of these devices, as observed in Figure 3b. The devices exhibit an endurance for up to 70 DC cycles without any electrical damage. As is known, endurance properties are commonly characterized by AC stimuli due to the lower damage compared to the DC. Some RS studies that have focused on SiO_x_-based devices have reported an endurance from 20 to 10^4^ cycles with AC stimuli [22,23]. As observed in Figure 3c, the mean values of current for LRS and HRS are about 1.26 × 10^−6^ A and 1.32 × 10^−12^ A for all the I-V cycles, indicating an ON/OFF mean ratio of ~10^6^, while a retention time larger than 10^4^ s was measured in these devices (see Figure 3d). ON/OFF ratios between 10^4^–10^5^ have been reported for SiO_x_-based devices [22,23].

As we can see in Figure 3c, there is a large variation of current for the LRS (ON) state, as compared to the HRS (OFF) state. This effect can be related with the conductive filament formation. As we know, the RS material in our devices is based on a SiO_2_/Si-NCs/SiO_2_ structure, where Si-NCs act as charge trapping nodes, supporting the formation of conductive filaments to obtain the LRS. It is strongly possible that with subsequent measurement cycles, after the breakdown of the first (main) conductive filament, other new—and different—conductive paths are formed in the system, which are likely to branch out from the undamaged nodes of the initial conductive path. As these branches break down under voltage sweeps in consecutive measurement cycles, one expects that some Si-NCs will end up insolated or blocked from further participation in the conductive filament formation, changing their resistance and therefore the current value at LRS.

**Figure 4:**
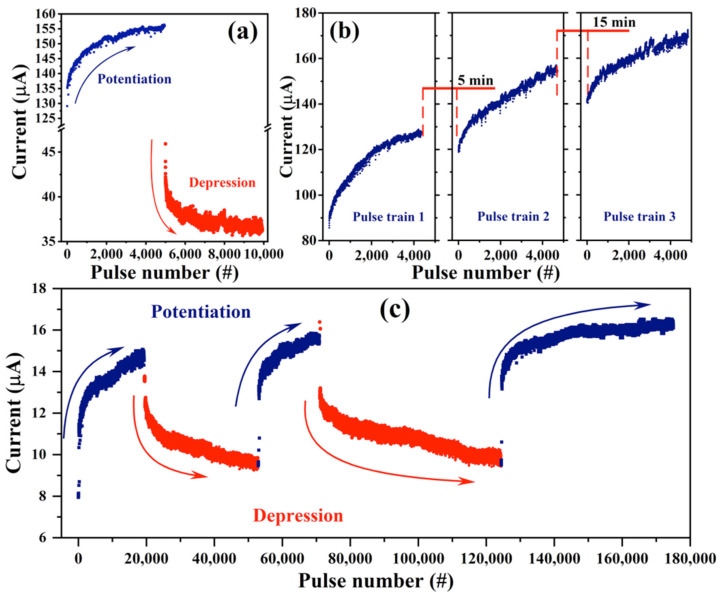
(**a**) Potentiation and depression processes in the SiO_2_/Si-NCs/SiO_2_ ML device; (**b**) current as a function of three different voltage pulse trains (write, −2 V) separated by 5 and 15 min; and (**c**) long-term potentiation (LTP/SET process) and long-term depression (LTD/RESET process) behavior under consecutive potentiating and depressing pulses.

The presence of different resistance levels is essential for the emulation of synaptic plasticity. For the analog synaptic device in neuromorphic computing, a gradual SET and RESET process is required to represent the synaptic weight value in artificial neural networks. Hence, the SiO_2_/Si-NCs/SiO_2_-based memristor device was analyzed with a pulse stimuli bias to observe its potential to mimic synaptic function. Potentiation and depression processes were achieved in these devices by applying a constant amplitude pulse train, as shown in Figure 4a. Identical pulses were applied with a pulse height/width of −2 V/150 ms to induce potentiation, whereas depression was achieved by applying identical pulses with a pulse height/width of 4 V/150 ms. As we can see, the current value gradually increases/decreases as the pulse number increases. The gradual increase/decrease of the resistance could be related to a charge/discharge effect within the ML structure. Then, to clarify the origin of the RS behavior, three different voltage pulse trains with a constant amplitude (write, −2 V/150 ms), separated by 5 and 15 min, were applied to the devices, as shown in Figure 4b. The device exhibits a gradual current increase from ~90 μA to ~125 μA, ~120 μA to ~155 μA, and ~145 μA to ~170 μA for the first, second and third pulse trains, respectively. As we can see, the last current value obtained at the end of each pulse train is kept, despite the device being unbiased for up to 15 min between the different pulse trains, which indicates its analog RS behavior. Finally, the devices showed a typical long-term potentiation (LTP/SET process) and long-term depression (LTD/RESET process) behavior under these consecutive potentiating and depressing pulses, as observed in Figure 4c. Therefore, this reliable potentiation and depression behavior indicates that our devices are promising candidates as electronic synapses for neuromorphic computing applications.

## 4. Conclusions

In summary, a memristor with digital and analog RS properties was proposed with the application of Si-NCs multilayer devices. These are promising first results due to the scarcity of similar studies in Si-NC devices in the literature. Additionally, thanks to the ability to stack a given number of layers, our devices have an extra degree of tuneability, allowing one to not only tailor parameters like the SET and RESET voltages but also to potentially adjust their analog behavior. Compelling future studies include a comparison of analog switching properties across devices with different numbers of Si-NC layers, as this could improve parameters such as the linearity of the analog switching process.

## Data Availability

Not applicable.
